# A Pre‐registered sticky mittens study: active training does not increase reaching and grasping in a swedish context

**DOI:** 10.1111/cdev.13835

**Published:** 2022-09-01

**Authors:** Linda van den Berg, Klaus Libertus, Pär Nyström, Janna. M. Gottwald, Victoria Licht, Gustaf Gredebäck

**Affiliations:** ^1^ Department of Psychology Uppsala University Uppsala Sweden; ^2^ Learning Research and Development Center University of Pittsburgh Pittsburgh Pennsylvania USA; ^3^ University of Milano‐Bicocca Department of Psychology Milan Italy

## Abstract

Several studies have previously investigated the effects of sticky mittens training on reaching and grasping development. However, recent critique casted doubts on the robustness of the motor effect of this training. The current study presents a pre‐registered report that aimed to generalize these effects to Swedish infants. Three‐month‐old infants *N* = 96, 51 females, mostly White middle class in Uppsala, received daily, parent‐led sticky mittens or observational training for 2 weeks or no training in 2019. Reaching and grasping abilities were assessed before and after training, using motion tracking and a 4‐step reaching task. Sticky mittens training did not facilitate successful reaching. These results indicate that beneficial motor effects of sticky mittens training did not generalize to this sample.

Learning to act and coordinate one's own body in relation to an ever‐changing environment is a difficult task to master. Crawling and walking are associated with massive amounts of experience. As toddlers learn to walk, they take more than 2000 steps and fall 17 times per hour. This experience extends over many months and locomotion is fine‐tuned and calibrated (Adolph et al., 2012). Cultural differences in the onset of proficient sitting can also be traced to daily activities that, over months, shape development and allow control over the many degrees of freedom associated with balancing the body (Super, 1976). A similar development can be seen for reaching and grasping. Foetuses, during the third trimester, perform actions that have many properties in common with reaching (Zoia et al., 2007) and this behavior is also observed in newborn infants (von Hofsten, 1991). Over the next 4–6 months, infants' daily and almost constant hand actions help fine‐tune reaching in order to make accurate hand movements that end in a grasped object. However, it is not until 8 to 12 months that infants' reaching actions are planned prospectively and these manual actions are well coordinated with a dynamic world (Gottwald et al., 2017).

In contrast to this protracted development, it has been proposed that a brief training procedure, the sticky mittens training, can facilitate and enhance manual motor behavior in a matter of days (Libertus & Needham, 2010; Needham et al., 2002) and this exposure will have long‐lasting effects on manual development more than 12 months later (Libertus et al., 2016). The sticky mittens paradigm has gained much popularity over the last 20 years. This paradigm includes the use of a parent‐guided interactive training game for approximately 10 minutes each day over a 2‐week period. The training is designed for infants who are not yet able to engage in successful reaching (i.e., a reach ending in a grasp of an object) by themselves. While wearing soft Velcro mittens, infants are encouraged to swat in the direction of objects covered in hard Velcro strips. Upon accidental or purposeful contact, these objects stick to the mittens, providing infants with simulated reaching experiences and opportunities to explore the object (Libertus & Needham, 2010; Needham et al., 2002). Previous studies have compared this training to a no training comparison group or an observational training group where the parent moves toys to the infant's hands and touches it.

The training is suggested to mimic the experiences of successful reaching (Needham et al., 2002) that would naturally emerge only around 5 months of age in western cultures (Von Hofsten & Ronnqvist, 1988). The act of repeatedly moving the hand forward with successful contact has been suggested to teach infants about the consequences of moving their own hand to an object (Needham et al., 2002). Infants are now able to bang the object on the table, shake the object, and closely explore the object visually or orally. From this exploration, infants gain information about the contingencies that these actions bring (Needham et al., 2002, 2017). The ability to pick up, manipulate, and explore the objects that are stuck to the mittens may provide opportunities for infants to learn about object properties (Needham et al., 2002), which may consequently affect attentional development (Libertus et al., 2016; Needham et al., 2002; Slone et al., 2018). The experience of reaching and grasping in the training might also provide infants with experiences of embodied agency (Delafield‐Butt et al., 2018; Delafield‐Butt & Gangopadhyay, 2013). Seeing themselves as intentional agents that attain goals has the potential to help infants understand that others are intentional agents that also attain objects (Gerson & Woodward, 2014a, 2014b).

Potentially accelerating the onset of successful reaching may have far‐reaching developmental consequences. Advanced motor capabilities relate to social cognition (Gredebäck & Daum, 2015; Gredebäck & Falck‐Ytter, 2015; Woodward, 2008), executive functions (Gottwald et al., 2016), language learning (He et al., 2015; Libertus & Violi, 2016; Wang et al., 2012), higher academic performance (Bornstein et al., 2013), and enhanced spatial processing and form perception (Möhring & Frick 2013; Schröder et al., 2019; Soska et al., 2010). If training effects are indeed reliable, then the sticky mittens paradigm might offer an interesting option to improve motor function in all infants, including infants at risk for Autism Spectrum Disorder (Libertus & Landa, 2014) and with diagnosed Cerebral Palsy (Nascimento et al., 2019).

In the last decade, training studies and practices have seen a large upswing in both adulthood and childhood. However, these studies have been criticized for the lack of generalization to new abilities, domains, and contexts (Barnes et al., 2016; Stojanoski et al., 2020). For instance, computerized attentional control training improves infant performance on the trained task (Wass et al., 2011), but attentional improvements do not extend to attentional and executive function abilities that were not trained (Ballieux et al., 2016). Although some have suggested training interventions may be more effective earlier in life (Wass et al., 2012), the long‐term effects of such interventions are unclear, and it remains unknown how much maintenance of the training is needed to facilitate wider transfer (Diamond & Ling, 2016). Consequently, it has been suggested that training practices may have little effect on general and long‐term development (Barnes et al., 2016; Stojanoski et al., 2020).

In a recent review, van den Berg and Gredebäck (2021) raised questions about the strength of the existing evidence linking sticky mittens training to gains in infant motor skills. On an overarching level, several studies report positive effects of sticky mittens training. However, when the details of this work are considered, it becomes less clear if motor benefits replicate across these studies. Two studies show increased reaching and grasping immediately after training (Libertus & Needham, 2010; Needham et al., 2002). One study does not demonstrate an effect immediately after training but instead reports a positive effect two months after the training (Wiesen et al., 2016). A forth study also fails to find a sticky mittens effect directly after training and even reports higher performance in a non‐sticky mittens control group (Williams et al., 2015), although it should be noted that this study made several modifications to the original training procedure. In a fifth study, Needham et al. (2017) demonstrated (in Experiment 1) that infants in the (normal) active sticky mittens condition showed the same amount of touching of a toy at both the pre‐test and post‐test, while infants in the observational condition touched the toy less at post‐test. This results in a significant difference between groups, but not in the same manner as reported in the two original studies with positive effects described above (Libertus & Needham, 2010; Needham et al., 2002).

With these inconsistencies in mind we believe it is important to replicate the sticky mittens paradigm. There is also a need to assess how infants respond to this training procedure in a non‐US sample. The current study aims to re‐examine the effects on infant motor development following two weeks of sticky mittens training in a Swedish sample. Previously, van den Berg and Gredebäck (2021) recommended more replications of the training and that future sticky mittens studies should be pre‐registered and analyze detailed motion tracking data (e.g., movement units, grasp aperture, and grasp preparation) in order to find clearer and more robust effects. This study follows all of these recommendations. In a pre‐registered, intent‐to‐treat, study, 3‐month‐old infants were assigned to one of three conditions: sticky mittens, observational, or no training. Although we only report on the motor effects of sticky mittens training, it is important to note that this study was a conceptual replication of the entire sticky mittens work. This includes the work on motor development, attention abilities, and social perception. Consequently, the ages in the current study were not matched to the ages in one specific sticky mittens study but to the age of the combined sticky mittens studies (i.e., 3 months of age). Whereas prior work has relied on video coding of reach and grasp proficiency, this is the first pre‐registered sticky mittens study to analyze detailed reach and grasp kinematics using high precision motion tracking technology in order to assess the detailed development of reaching and grasping prior to and following sticky mittens training. Our hypothesis, at the time of pre‐registration, was that infants in the sticky mittens condition (but not the other conditions) would show increased motor performance following training. Specifically, we predicted a decrease in movement units (von Hofsten& Lindhagen, 1979), an increase in grasp aperture, and an earlier grasp initiation before object contact from pre‐test to post‐test (von Hofsten & Rönnqvist, 1988). Grasping abilities typically emerge 4 weeks after reaching abilities emerge (von Hofsten & Lindhagen, 1979). However, if the training were found to be successful, we would expect that grasping abilities emerge sooner than would be expected in typical development. This study was planned, pre‐registered, and conducted, but not analyzed, prior to the review by van den Berg and Gredebäck (2021) that expresses concern about the presence of a sticky mittens effect in the motor domain.

## METHODS

### Participants

Ninety‐six participants (51 females) from the Uppsala, Sweden, region visited the lab at the age of 3 months (see Table 1 for demographics). Infants were selected via the Uppsala university infant database, which infants entered through a letter that was sent out to parents at 2 months. This was followed‐up by a phone call at 3 months in which information about the study was provided and parents were asked to participate. Data were collected in a university city (February–October 2019) with high levels of education. Infants came from households with a medium to high socio‐economic status and most parents visiting the lab typically had academic backgrounds. Although there was no official record of ethnicity, the participants were predominantly White, with only a few exceptions (African, South Asian, and Middle Eastern). The exact number of non‐White families (and other details) is unknown as the Uppsala Child and Baby Lab does not allow an official examination of these type of data. Parents received 480 days of parental leave per child, which were to be taken out by both parents. The pre‐registered sample size rationale was based on Libertus and Needham (2010), but was confirmed by a power analysis prior to data collection but outside of the pre‐registration. An a priori power analysis was conducted in G*Power (Faul et al., 2007). The power analysis (assuming two‐tailed *alpha =* .05, an *F*‐statistic, and an effect size of *d =* 0.40) indicated that a total sample of 57 infants, with three equally sized groups of 19 infants in each condition, would yield sufficient power of .80. The sample size was set to 96 infants in order to increase power and maintain sufficient power to account for potential dropouts. Participants were randomly allocated to one of three training conditions: sticky mittens, observational, and no training. In total, two participants (one in sticky mittens and another in the no training condition) were born prematurely. Exclusion of these participants did not change the results. One participant did not complete the training (observational condition) but returned for a second visit. Two participants did not return for a second visit (one sticky mittens condition, one observational condition). The data of two participants (one sticky mittens condition, one observational condition) were lost due to technical difficulties. Following our pre‐registered intent‐to‐treat protocol, no infants were excluded from the study and analyses were conducted without replacing missing observations. Parents gave their written consent before participation and received gift vouchers worth approximately €10 for each visit to the lab. In accordance with the 1964 declaration of Helsinki, the study obtained ethics approval from the local ethics review committee.

**Table 1:** Summary of participant characteristics and number of trials and reaches in all three conditions

Condition	*n*	Age pre‐test (weeks)	Age post‐test (weeks)	Number of females	Parental education (university) (%) ^a^	Total training duration (minutes)	Total Trials	Total reaches pre‐test	Total reaches post‐test	Average reaches
Sticky mittens	32	13.36 (1.49)	15.60 (1.44)	15	69	89.57 (41.56)	505	83	124	4.06 (2.31)
Observational	32	13.39 (1.50)	15.58 (1.63)	19	54	94.93 (41.73)	522	74	114	4.00 (1.95)
No training	32	13.14 (1.39)	15.12 (1.70)	17	83	‐‐	503	84	133	4.09 (2.10)

*Note:* Standard deviations are included in parentheses.

^a^ Parental education of both parents was assessed, with answers ranging from elementary school to university. The percentage of parents with a university degree was reported. Parental education did not differ between conditions, *p* > .05.

## DESIGN & PROCEDURE

### Training

Between 3 months and 3.5 months of age, participants received one of three types of home‐based training: sticky mittens training, observational training, or no training. The sticky mittens condition was a daily, 2‐week training in which participants interacted with objects using sticky mittens, while the observational training was a daily, 2‐week training in which participants observed the parent move an object in front of the participant. Participants in the no training condition did not take part in any training and visited the lab for the pre‐ and post‐tests.

In both the sticky mittens and observational condition, infants trained with felt mittens covered with Velcro loop on the palmar side of the hand (Needham et al., 2002). They received three sets of Duplo blocks (4.5 x 4.5 x 4.5 cm) with either patches of Velcro hook (sticky mittens condition) or patches of electrical tape of a similar size (observational condition) stuck to the blocks. All blocks rattled when shaken as this has been suggested to increase effectiveness of the sticky mittens training (Needham et al., 2017).

**Figure 1:**
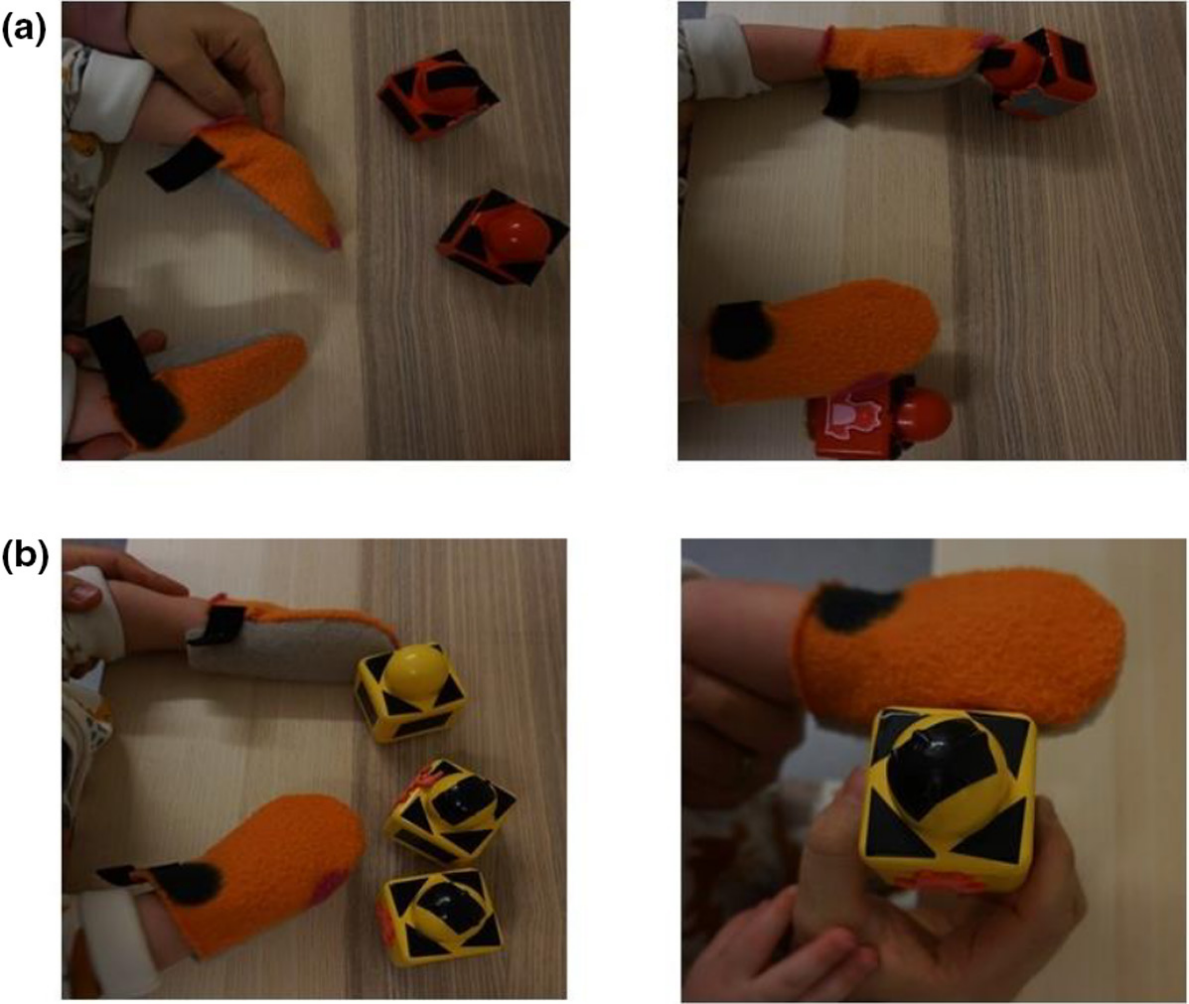
Examples of the sticky mittens training (a) where the infant swats the blocks that stick to the mitten. In the observational training (b), the parent moves the block to the infant's hand and does not stick to the mitten.

In the sticky mittens condition (see Figure 1) parents were instructed to sit at a table with their infant on their lap, making sure to provide proper waist and trunk support and that the infant could comfortably reach for the objects. After putting mittens on the infants' hands, parents placed the first set of objects within the infants' reaching space. Parents demonstrated the stickiness of the mittens by lifting, shaking, and moving the object toward the mitten and letting it stick. Afterwards, the training started by placing one single object on the table and subsequently attracting attention to it verbally, by pointing to the object, or by tapping the object on the table. Parents did not help the infant reach for the object. After successful object‐contact, parents praised the infant and allowed the infant 10 s to explore the object that was stuck to the mitten. This process was repeated for approximately 3–4 min, after which the sequence was repeated with the set of two and three blocks.

The observational condition (see Figure 1) was identical to the sticky mittens condition, except that infants did not independently act on the objects. The parents placed one object in the infants' reaching space, drew attention to the object by pointing, and lifted the object to the infants' eyes. Afterwards, the object was tapped on the table, lifted back to eye‐level, and moved to the mitten‐covered hand. This process was repeated for approximately 3–4 min while alternating the hand that touched the object. Afterwards, the parent repeated the process with the other sets. In both the sticky mittens and the observational conditions, parents filled in a logbook to record training frequency and duration after each training event. To ensure proper execution of the training, caregivers received a demonstration of the training, written instructions, and a small group joined video conferences in which a trained experimenter (author K.L.) observed a training session. Eight participants from the sticky mittens condition and six participants from the observational condition attended the video conferences. Parental training procedures in this small selection of participants did not raise concerns that parents may have misunderstood the training instructions.

### Assessment

This study was a longitudinal training study in which participants visited the lab at three ages: 3 months, 3.5 months, and 10 months, with the home training occurring between 3 and 3.5 months. The current study reports the effects of sticky mittens training on the 4‐step reaching task, the kinematic reaching task, and general reaching development between the visits at 3 (before training) and 3.5 (after training) months of age. Given that van den Berg and Gredebäck (2021) called for caution when interpreting the sticky mittens work, this study only compares manual performance at 3 and 3.5 months – before and after the 2‐week training period. This approach should maximize any potential training effects and enables us to focus our discussion on the core sticky mittens effect. At both the pre‐test and post‐test lab visits, participants first completed a 4‐step reaching task (taken from Libertus and Needham, 2010) and then a kinematic reaching task (new for the current study). The 4‐step reaching task measured reaching and grasping behaviors by incrementally moving an object closer to the infant until the object was placed in their hand. The object was an easy‐to‐grasp, ring‐shaped, teething rattle (diameter = 10 cm), similar in shape to the rattle used in Libertus and Needham (2010). While an infant sat at a table, on the parent's lap, an experimenter incrementally placed the object in 4 different locations: out of reach, at the edge of the reaching space, in the reaching space, and in the hand. The infants' reaching space was defined during pilots, which indicated that approximately 15 cm was the maximum position where infants could still grasp the toy. Each step lasted 30 s and started with the experimenter attracting attention to the toy by saying ‘Hey *infant's name*’ and shaking the object.

**Figure 2:**
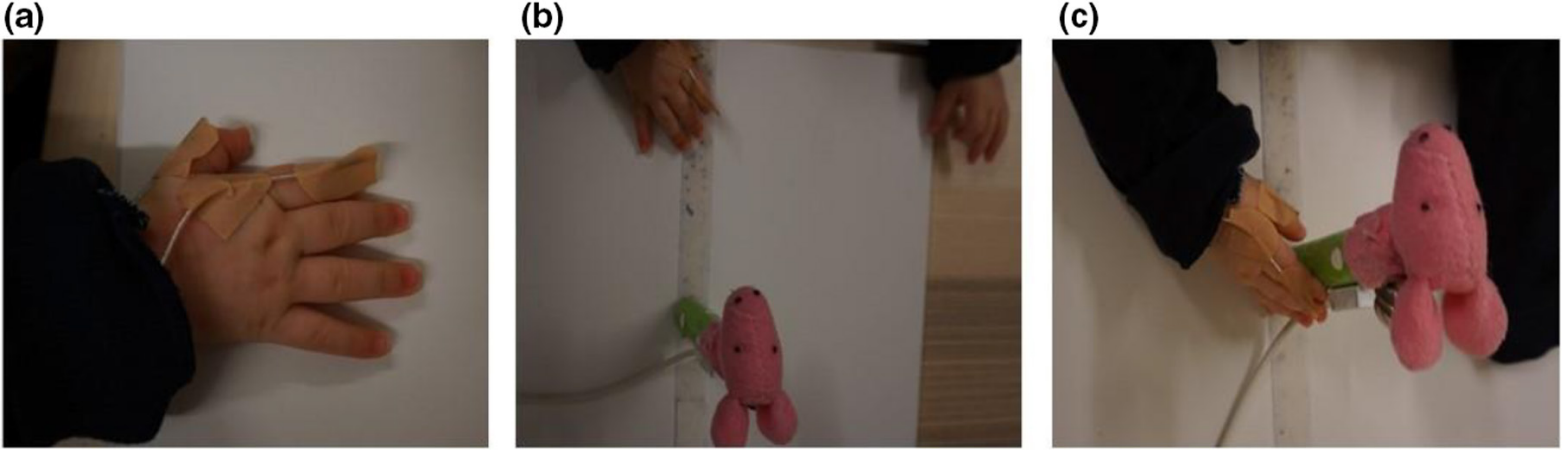
Example of the reaching task. Motion tracking sensors (a) were taped to the thumb and index finger. Infants' tracked hand was kept as close to the body as possible at the start of the trial (b). A reach ended when the infant touched the object with their tracked hand (c).

The kinematic reaching task was created to measure reaching and grasping of a stationary object. This task enabled an examination of infants' reaching and grasping abilities for an easy‐to‐obtain object. Objects in this task were two cylindrical toys (height = 12 cm, diameter = 1.5 cm) decorated with different colored tape (blue or green), a finger puppet (duck or pig), a bell to attract attention, and a motion tracking sensor on each object to track the position of the object (see Figure 2). Inside the objects were rattle inserts to attract attention upon touch or when shaken. Objects were placed on a white board (33 x 39 cm). Infants sat on their parent's lap at a table, while an experimenter sat on the other side at a distance of 80 cm. An object was placed on a rectangular board and the experimenter slid the board forward towards the infant until it was within reaching distance, approximately 15 cm from the participant. The sliding of the board marked the start and end of each trial, both in the motion tracking data as in the video recordings. Immediately after the board arrived at the infant, the trial started. At the start of a trial, the infants' hands were at the edge of the table, to ensure a similar distance for each infant. Fussy infants who did not keep their hands here were repositioned such that they always started reaching from the edge of the table. During the trial, parents were allowed to encourage reaching, by shaking the object or pointing to attract attention, without helping the infant reach for the object. When the infant reached for and grasped the object, the object was placed back on the board and infants were encouraged to repeatedly reach for the object until the end of the trial. The trial ended after 30 s, after which the experimenter slid the board back to mark the end of the trial. In total, there were eight trials to obtain as many reaches as possible without exhausting the infant.

After these tasks, several eye tracking tasks were used to assess social perception and attention: face preference (Libertus & Needham, 2011), teleological reasoning (Gredebäck & Melinder, 2010), action prediction (Falck‐Ytter et al., 2006; Green et al., 2016), and free‐viewing (van Renswoude et al., 2019). These tasks will be reported in a subsequent report. After each visit to the lab, families filled in an on‐line version of the Early Motor Questionnaire (general motor development, EMQ; Libertus & Landa, 2014).

### Pre‐registration

This study was pre‐registered at the Open Science Framework prior to data collection (osf.io/hx6ma). All data were handled according to an intent‐to‐treat protocol. The 4‐step reaching task consisted of attention and exploration steps (step one and four) and manual steps (step two and three). The current study deviated from the pre‐registration in that only the manual steps of the 4‐step reaching task were reported to facilitate a focused discussion of the manual effects of the sticky mittens training. For more information on pre‐registered and additional exploratory analysis see below.

### Data Preprocessing

Reaching and grasping were recorded using motion tracking technology (Ascension trakSTAR, Waterloo, Canada, 80 Hz). This technology tracked the movements of the hand, more specifically the thumb and index finger. Infants wore a sweater with two sewn‐in motion tracking sensors to streamline sensor placement. The sensor wires came out through the hand opening and were taped to the right thumb and index finger with surgical tape (see Figure 2). Four motion tracking sensors captured reaching behavior, one sensor each (2 mm) on the thumb and index finger and one sensor each (7.9 mm) on the objects. A video camera recorded the trials while the motion tracking system recorded participants' movements.

Using frame‐by‐frame video coding in the Qualisys Track Manager software (version 2019.2; Gothenburg, Sweden), the start and end of the reaches were manually coded in the video recordings of the task. To initiate a trial the experimenter moved a board, containing the target toy, forward towards the infant (see Figure 2). To facilitate preprocessing, the video frame before the start and end of the trial (the forward and backward movements of the board) were coded. A reach was defined as any continuous movement in a uniform direction towards the object, starting at least 5 cm from the object, which ended in physical contact with the object, within a trial. The video frame before the start of this movement was coded as the start of the reach and the frame before the first contact with the toy was coded as the end of the reach. Although more reaches were possible within a trial, only the first reach in each of the eight trials was counted as a valid reach to examine the quality of the reach rather than the frequency of reaching. Many of the subsequent reaches might not be goal‐directed, forward extensions, which introduces subjective assessment of valid reaches. However, the first reach provides an objective assessment of reaches in the motion tracking data.

The motion tracking data were preprocessed in the analysis tool Timestudio (version 3.19; Nyström et al., 2016) for MATLAB (version 9.2). The motion tracking system was set to sample data at 80 Hz. Following the preprocessing steps of previous research (Gottwald et al., 2017; Grönqvist et al., 2011), the data were filtered using a five‐sample moving average filter on the x‐, y‐, z‐coordinates of the position data. Subsequently, 3D velocity was calculated and filtered with a five‐sample moving average filter. The 3D velocity was calculated by using Pythagoras' theorem using the equation velocity3D = sqrt(velocityX^2^ + velocityY^2^ + velocityZ^2^).

**Figure 3:**
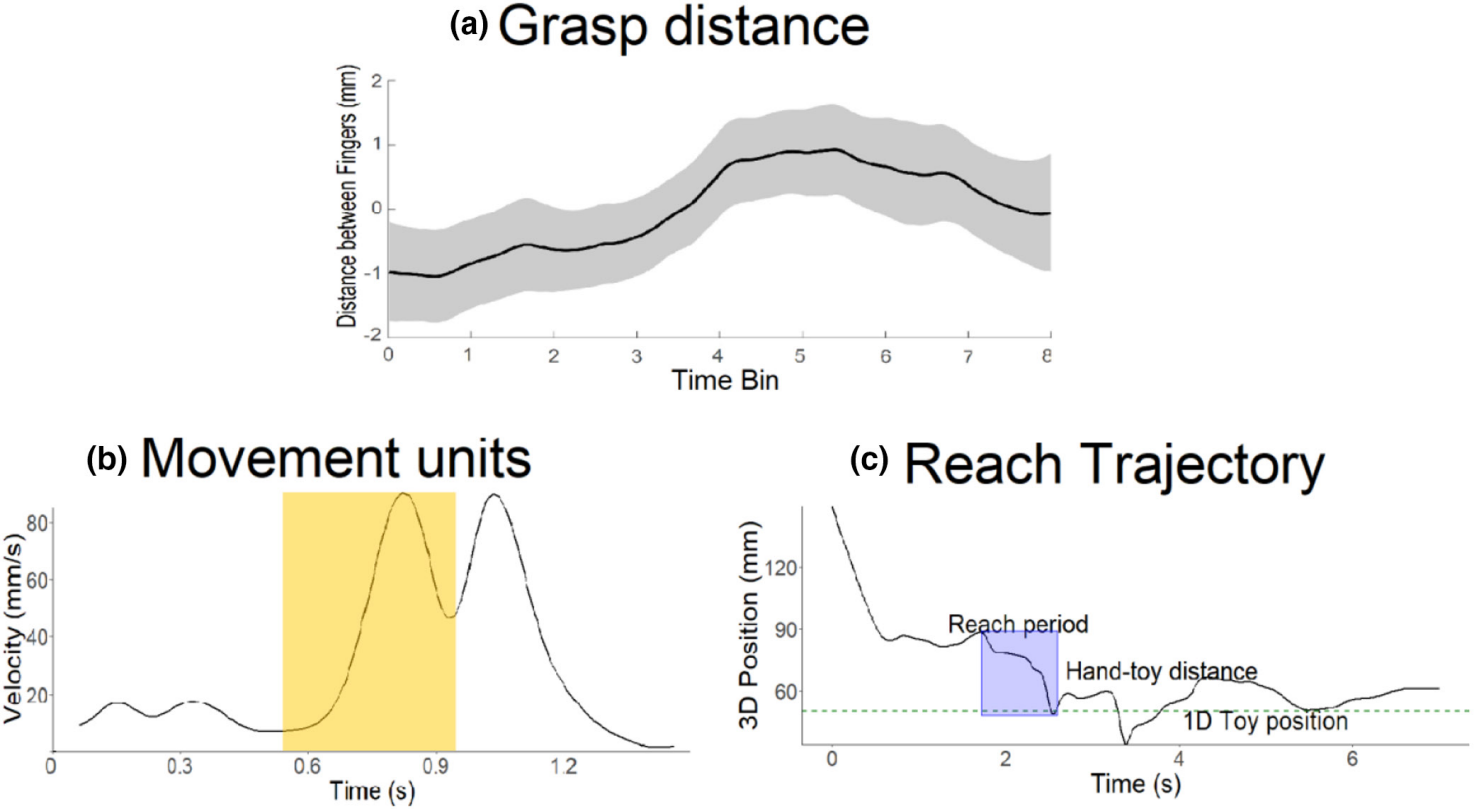
a) The distance between the index finger and thumb over an 8‐second period at pretest. Each bin corresponds to a second. Baseline corrected for illustration. b) Example of the number of movement units in a reach of one participant. The shaded area highlights an individual movement unit with one acceleration and one deceleration event. c) Example of the reach selection process of a trial. The black solid line represents the position of the movement of the hand. Descending hand position indicated that the hand moved toward the object while ascending position indicated that the hand moved away from the object. The green dotted line represents the position of the object. The reach period indicates that the hand (black line) moved in a uniform direction until it reached the object (green line).

The kinematic reaching task measured reaching and grasping via number of first reaches, reach duration, number of movement units per second, grasp aperture, grasp aperture before touch, and grasp aperture after touch (for illustrative examples see Figure 3 and Table 2). The number of first reaches were based on the video recordings and the timestamps of the start and end of each reach in the video recordings were used to identify the reaches in the motion tracking data. Each duration was defined as the time between the extension of the hand (start of the reach) and hand‐object contact (end of the reach). The motion tracking timestamps of the start and end of each individual reach were used to calculate the duration of each individual reach. To stay close to Needham et al. (2002), the number of reaches were defined as the number of trials in which a first reach was coded. To assess whether the reach becomes smoother (i.e., whether it contains less movement units) after training, a movement unit was defined as an acceleration followed by a deceleration phase in the velocity profile of a reach (von Hofsten, 1991). Movement units were detected using the following parameters. The minimum distance required between a movement unit peak and the neighbouring minima was set to 2.3 cm. If the distance between a peak and a neighbouring peak was smaller than one sample, this smaller peak was not included as part of a movement unit. No minimum height was set for the height of a movement unit peak, nor was there a requirement for how much larger a peak should be compared to the nearby peaks. To account for noise and movement units that are too small to be biologically plausible, movement units that were one sample or smaller were merged into one movement unit (see Figure 3; Gottwald et al., 2017). The number of movement units per second were calculated as the measure. The number of movements units per second for the first reach of all trials were averaged to create a single measure.

**Table 2:** Infants' performance on all dependent variables separated by time of assessment

Measures	Definition	Pre‐test	Post‐test
Time spent reaching and grasping (%)	Time spent reaching, grasping, lifting, and exploring	4.01 (9.90)	9.70 (15.35)
Number of reaches	Number of reaches over all trials.	2.51 (2.18)	4.03 (2.56)
Reach duration (s)	The time from the start of the reach until toy contact.	0.59 (0.40)	0.73 (0.42)
Movement units per sec	The number of acceleration and deceleration phases in a single reach.	3.32 (0.84)	3.17 (0.49)
Grasp aperture (cm)	The distance between the index finger and thumb.	2.84 (4.4)	2.56 (2.75)
Predictive grasp aperture (cm)	The average distance between the index finger and thumb before toy contact.	9.76 (2.28)	8.95 (2.13)
Reactive grasp aperture (cm)	The average distance between the index finger and thumb after toy contact.	7.83 (2.03)	6.98 (2.20)
Reaching EMQ		−31.27 (10.36)	−28.79 (11.85)

*Note*: Average performance is reported with standard deviations in parentheses.

To assess whether infants grasp objects with closed or open hand (von Hofsten, 1984), the peak distance between the index finger and thumb, at the moment of touch, was measured (von Hofsten & Rönnqvist, 1988). The distance between the motion tracking data of both fingers was extracted. The distance corresponding with the timestamp of the end of the reach (i.e., object‐touch) was taken as the grasp aperture. This measure was calculated for the first reach in each trial and averaged to create one measure of grasp aperture. It is important to note that the grasp aperture measure, on its own, does not allow us to identify proficient grasping as it is merely the extent of hand opening upon object‐contact. However, if this aperture changes from pre‐test to post‐test, this would indicate grasping improvement, as has been demonstrated when infants naturally improve their grasping (von Hofsten & Rönnqvist, 1988).

Finally, we examined whether infants started preparing their grasp in a predictive manner, before object‐touch, or in a reactive manner, occurring after object‐touch (von Hofsten & Rönnqvist, 1988). To this end, the extraction of motion tracking data was extended beyond the time window of the reach. The distance between the index finger and thumb was calculated in the second before touch and the second after touch, see Figure 3. This calculation was performed for the first reach in each trial and then averaged to create a single measure. Differences between conditions in the average grasp distance in the second before touch would indicate that infants in one condition start grasping the object during the reach and before their hand arrives at the object, compared to other conditions. Similar differences in the second after touch would indicate that infants start preparing their grasp only after their hand arrives at the object.

A trained observer coded motor abilities in the 4‐step reaching task, using Datavyu (Datavyu Team, 2014), following the coding scheme in Libertus and Needham (2010). The following manual behaviors were coded in the manual steps: visually guided arm extensions toward the toy (reaching), toy contact (touching), lifting one corner of the toy off the table (grasping), lifting the toy, and exploring the toy. The durations for each behavior were coded and combined to create one measure of reaching and grasping. The dependent variable was the percentage of time an infant spent reaching and grasping.

### Confirmatory hypothesis testing

Initially, the pre‐registered analyses were Linear Mixed Models on the time infants spent reaching and grasping, the number of movement units, grasp aperture, and predictive and reactive grasp aperture as dependent variables, with time and condition as independent variables. However, the Linear Mixed Models would require a more elaborate and complex report (see Supplementary Information for these results). To facilitate communication of the results and to prevent interference with the interpretation of this study, the more common and easy‐to‐interpret equivalent of these analyses were conducted. Both analyses demonstrated nearly identical results. Three (condition) x 2 (time) mixed analyses of variance (ANOVAs) were conducted on the same dependent variables. Both main effects and interactions were examined.

Following the intent‐to‐treat protocol, all analyses were performed using all participants and all data. If data points exceeded 3 SD above or below the mean, analyses were repeated without these individual data points to check for robustness of results. Three infants from the sticky mittens condition (one at pre‐test), one infant from the observational condition (at pre‐test), and one infant from the no training condition (at pre‐test) were considered outliers on the grasp aperture measure. Two infants from the sticky mittens condition (post‐test) and one infant from the observational condition (pre‐test) were outliers on the 4‐step reaching and grasping measure. Excluding these data points did not change the results. All analyses were carried out in R (R Core Team, 2018).

### Exploratory analyses

In addition to the detailed kinematic measures above, it was important to examine measures that are often reported in other sticky mittens work. Therefore, to further examine the effects of training on reaching, we conducted 3 (condition) x 2 (time) ANOVAs on reach duration and the number of reaches. All analyses were conducted on the full data (i.e., all first reaches in all trials) and repeated with removal of outliers to check the robustness of the results. On the reach duration variable, one participant from the sticky mittens condition was an outlier (at pre‐test). Exclusion of outliers did not change the results.

In order to examine the effect of training on reaching and grasping behaviors that are not connected to the two tasks in this study, a composite score of the EMQ was computed. This early motor questionnaire measured general motor development (Libertus & Landa, 2013). The composite score consisted of only items related to reaching and grasping abilities. Therefore, this approach is more focused (and potentially more powerful) than comparing full EMQ sub‐scales. This score consisted of two items from the gross motor scale, 19 items from the fine motor scale, and 10 items from the perception abilities scale. We conducted an additional 3 (condition) x 2 (time) ANOVA on a composite score representing reaching and grasping abilities in the EMQ. This analysis was repeated with the separate EMQ scales to check for differences in results and these results are reported in the Supplementary Information.

In order to examine whether training effects were influenced by the amount of training, a potential dose–response relation was analyzed. Pearson's correlations were conducted on training durations and the abovementioned variables. These preliminary dose–response analyses are reported in the Supplementary Information. We also repeated the main analyses on the data including only infants who had completed at least 60 minutes of sticky mittens or observational training ‐ this did not change the results (see Supplementary Information).

Previous work indicated that long reaches were characterized by more movement units, while short reaches contained less movement units (Mathew & Cook, 1990).To understand general reaching and grasping abilities regardless of condition, we conducted correlation analyses on the previously mentioned variables. We conducted Pearson's correlations at pre‐test and post‐test to examine this relation. Using a median split, reach durations for individual reaches were divided into proficient (fast) and less proficient (slow) reaches for analysis. Pearson's correlations were conducted on reaching proficiency and grasp ability at pre‐test and post‐test.

To examine the characteristics of reaching and grasping in this period of infancy, regardless of training or time of assessment (pre‐test and post‐test), individual differences in reaching were described.

In order to examine the stability of the measures in this study, Pearson's correlations were conducted on reach durations, number of movement units, and EMQ reaching from pre‐test to post‐test, collapsed over conditions to examine the stability of reaching from pre‐test to post‐test. To examine the stability of reaching within trials, Pearson's correlations were conducted on reach durations and number of movement units of the first two reaches of each infant.

## RESULTS

Collapsed over all conditions, pre‐ and post‐tests, infants reached for the objects on 4 out of 8 trials (*M* = 4.05, *SD* = 2. 11, range = 1–8), which resulted in 612 analyzed reaches. For more information related to the separate conditions, see Table 1.

### Confirmatory Analyses

#### Time Spent Reaching and Grasping

The time infants spent reaching and grasping increased from pre‐test to post‐test, *F* (1,183) = 8.38, *p* = .004, *ɳ*_*p*_^*2*^ = 0.04 (see Figure 5). Training condition did not affect the time they spent reaching and grasping, *F* (2, 183) = 0.50, *p* = .61, *ɳ*_*p*_^*2*^ < 0.001. There was also no interaction effect between condition and pre‐ vs post‐test, *F* (2, 183) = 1.44, *p* = .24, *ɳ*_*p*_^*2*^ = 0.02 These results suggest no increase in successful reaching following two weeks of motor training in either of the three groups compared here – contrary to the results reported in Libertus and Needham (2010). These findings confirm the motion tracking findings below.

### Number of Movement Units

The number of movement units per second did not change from pre‐ to post‐test**,** *F* (1, 182) = 0.30, *p* = .59, *ɳ*_*p*_^2^ < 0.001. There were no differences in movement units per second between the three conditions, *F* (2, 182) = 1.39, *p* = .25, *ɳ*_*p*_^*2*^ = 0.01 and no interaction between condition and pre‐ vs post‐test, *F* (2, 182) = 0.08, *p* = .92, *ɳ*_*p*_^*2*^ = 0.002. Although equal variances were not assumed, a robust ANOVA (Mair & Wilcox, 2019) did not change the results. Infants in all conditions had approximately the same movement units from pre‐test to post‐test and the sticky mittens condition did not alter the velocity profile of the reach more than controls.

#### Grasp Aperture

Infants did not change their grasp aperture at object‐touch from pre‐ to post‐test, *F* (1, 182) = 0.004, *p* = .95, *ɳ*_*p*_^*2*^ = 0.004. There were no differences in grasp distance at object‐contact between the three training conditions, *F* (2, 182) = 1.02, *p* = .36, *ɳ*_*p*_^*2*^ < 0.001. There was also no interaction between condition and pre‐vs post‐test, *F* (2, 182) = 0.92, *p* = .40, *ɳ*_*p*_^*2*^ = 0.01. The sticky mittens training did not facilitate grasp aperture at object‐touch.

#### Grasp aperture before touch

Infants in the three conditions did not differ in their average grasp aperture before they touched the object, *F* (2,143) = 0.12, *p* = .89, *ɳ*_*p*_^*2*^ = 0.002. The grasp aperture did not change from pre‐test to post‐test, *F* (1, 143) = 1.30, *p* = .26, *ɳ*_*p*_^*2*^ = 0.009. There was no interaction between condition and pre‐vs post‐test, *F* (2, 143) = 0.17, *p* = .85, *ɳ*_*p*_^*2*^ = 0.002. These results indicate that there were no differences between the three conditions in the preparation of the grasp before the infants touched the object. Sticky mittens training did not facilitate infants' ability to prepare for a grasp.

#### Grasp aperture after touch

Infants decreased their grasp aperture from pre‐test to post‐test, *F* (1, 143) = 4.96, *p* = .03, *ɳ*_*p*_^*2*^ = 0.03. The distance between the index finger and thumb was smaller at post‐test after the infant had already touched the object. Infants in the three conditions did not differ in the grasp aperture after they touched the object, *F* (2, 143) = 0.05, *p* = .95, *ɳ*_*p*_^*2*^ < 0.001. There was no interaction between condition and pre‐vs post‐test, *F* (2, 143) = 1.36, *p* = .26, *ɳ*_*p*_^*2*^ = 0.02. This indicates that sticky mittens training did not facilitate infants' ability to initiate a grasp after touching the object.

### Exploratory Analyses

#### Number of reaches

**Figure 4:**
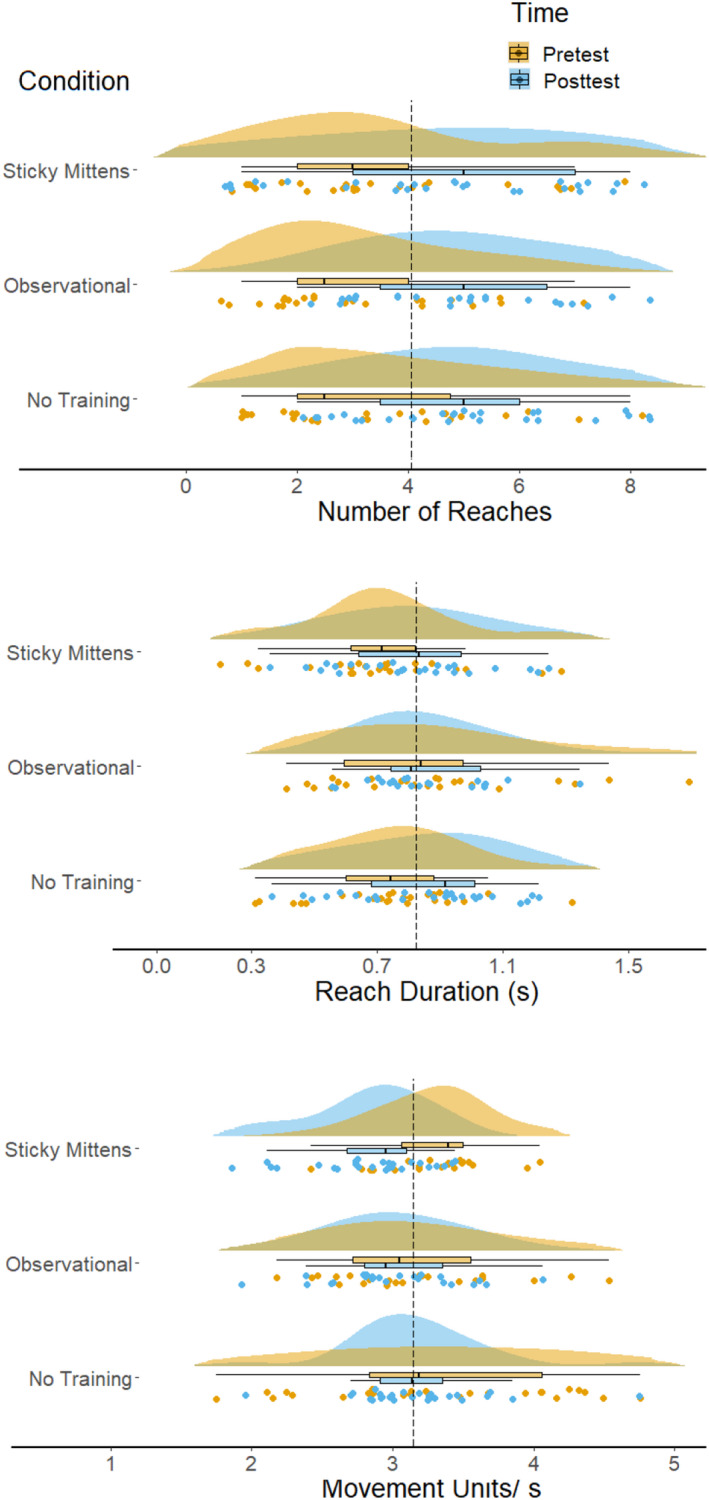
The number of reaches (top panel), reach duration (middle panel), and movement units (bottom panel) in all three condition. The black line corresponds to the overall mean.

The number of reaches increased from pre‐test to post‐test, *F* (1,182) = 18.82, *p* < .001, *ɳ*_*p*_^*2*^ = 0.09 (see Table 2 and Figure 4). Training condition did not affect the number of reaches, *F* (2,182) = 0.27, *p* = .77, *ɳ*_*p*_^*2*^ = 0.003. There was also no interaction effect between condition and pre‐ vs post‐test, *F* (2,182) = 0.03, *p* = .970, *ɳ*_*p*_^*2*^ < 0.001 These results showed that infant reaching improved from pre‐test to post‐test, as indicated by the number of reaches, but that it was not affected by training.

**Figure 5:**
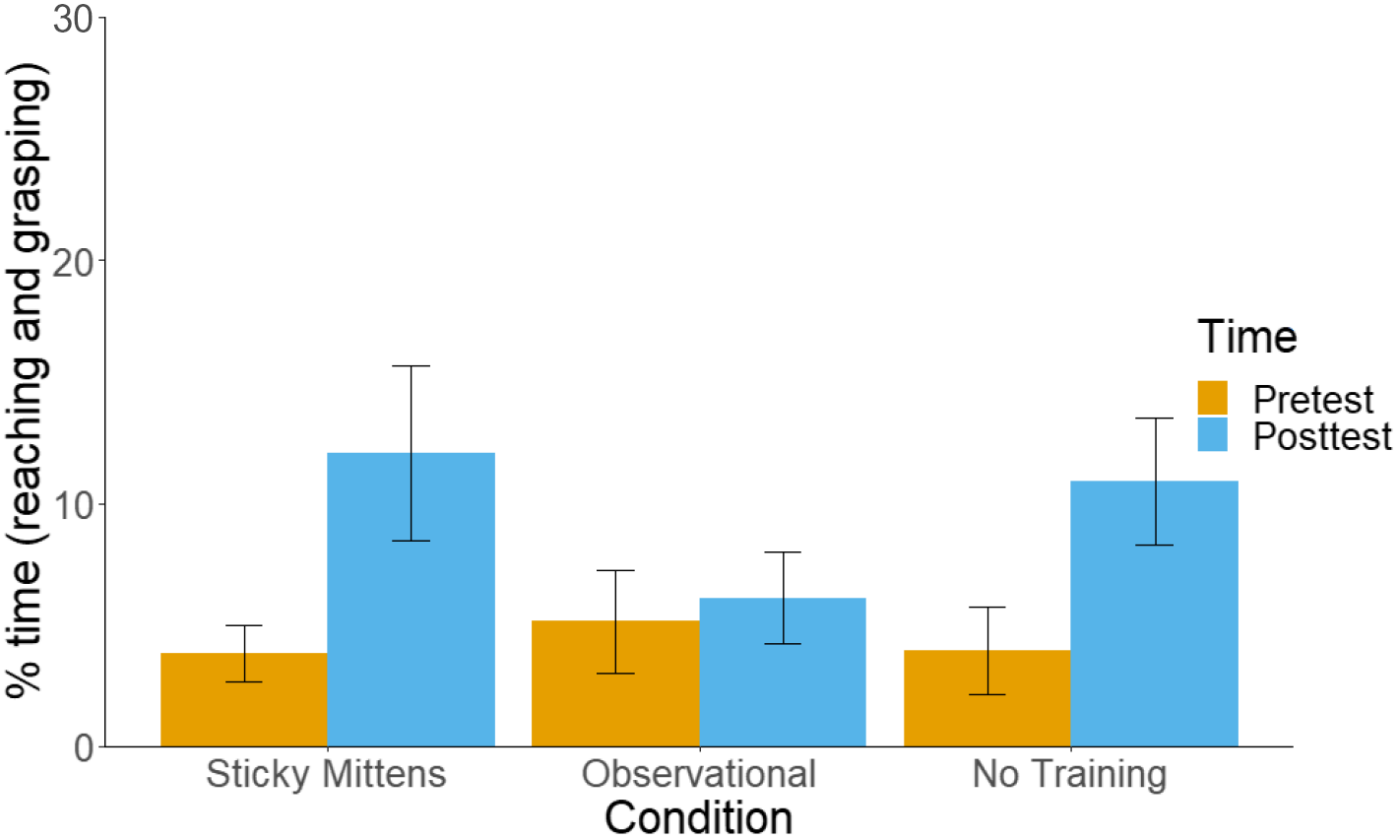
The percentage of time infants spent reaching and grasping during the manual steps of the 4‐step reaching task. Error bars represent Standard Error of the Mean.

**Figure 6:**
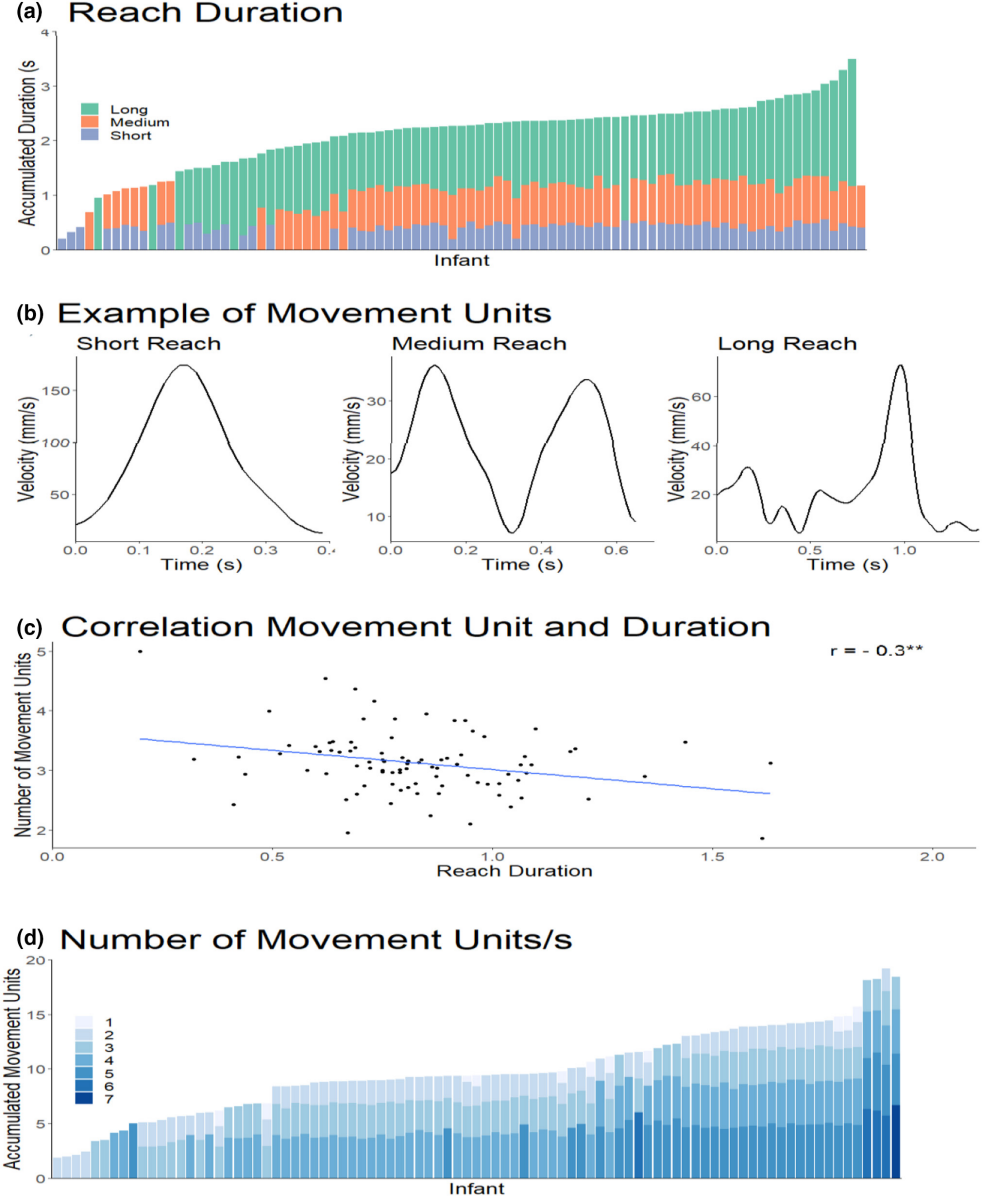
Individual reaching at pre‐test. a) illustrates the accumulated duration for short, medium, and long reaches per individual participant. Each section in the bar indicates the total duration over all reaches in that category. b) illustrates the smoothness (number of movement units) of a short, medium, and long reach of a participant. c) illustrates the correlation between reach duration and the number of movement units. d) illustrates the accumulated number of movement units per individual participant. Each section in the bar indicates the total number of movement units over all reaches in that category. ** p < 0.01.

#### Reach duration

Reach durations increased from pre‐test to post‐test, *F* (1,182) = 5.18, *p* = .024, *ɳ*_*p*_^*2*^ = 0.03. Training condition did not affect reach durations *F* (2,182) = 0.08, *p* = .92, *ɳ*_*p*_^*2*^ = 0.001. There was no interaction effect between condition and pre ‐vs post‐test, *F* (2,182) = 0.68, *p* = 0.51, *ɳ*_*p*_^*2*^ = 0.01. These results indicated that infants' reach durations changed from pre‐test to post‐test, but that this increase was the same for all infants. Sticky mittens training did not facilitate an increase in reach durations (Figure 4 and Supplementary Information for pre‐test and post‐test performance).

#### EMQ general reach development

The composite score for reaching did not increase from pre‐test to post‐test, *F* (1, 141) = 2.14, *p* = .15, *ɳ*_*p*_^*2*^ = 0.02 and training did not affect the reaching score, *F* (2, 141) = 2.00, *p* = .14, *ɳ*_*p*_ *2* = 0.03. There was no interaction between the three conditions and pre‐ vs post‐test *F* (2, 141) = 0.35, *p* = .70, *ɳ*_*p*_^*2*^ = 0.005, suggesting that reaching behaviors, as measured by the EMQ, did not change as a consequence of active sticky mittens training.

#### Age

Given that the age of infants in this study was matched to the average age in prior sticky mittens studies, and not to an age in a particular paper, it is conceivable that there are local age differences between studies that can account for variations in results. In order to account for this, we ran new analyses in which age (in days) was added as a covariate. The new analyses replicated findings reported above and did not demonstrate any interactions with age (all *p*s *> .05*), suggesting that the ages in this study did not affect the results.

#### Individual differences

The significant changes from pre‐test to post‐test in almost all dependent variables called for a further examination of this change over time. The number of movement units per second correlated positively with reach durations at pre‐test, *r* (69) = .28, *p* = .02, but not at post‐test, *r* (75) = .18, *p* = .12. This indicated that infants' fast reaches consisted of fewer movement units at pre‐test, while slower reaches consisted of more movement units. A partial correlation revealed that this relation was influenced by the peak velocity of the movement units, *r* (64) = −.38, *p* = .002. This is in line with the idea that long reach durations are characterized by movement units with lower peak velocities (Berthier & Keen, 2006). Reach proficiency and grasp ability did not correlate, nor was there stability from pre‐test to post‐test or within participants on reach duration, the number of movement units, or the EMQ reaching score (*r* ‐values ranged from −.21 to .23).

To map the individual differences in the reaching data, the accumulated reach durations were illustrated in Figure 6, including the number of movement units in a reach for individual participants. To this end, reach durations were split into three categories, which resulted into: short (<0.5 s), medium (0.5–0.8 s), and long (0.8–2.8 s) reaches. Figure 6 provides information about how reaches look in terms of duration and movement units around the age of 3 months and whether this is the same in each reach. When pre‐test and post‐test data were collapsed, there was a correlation between reach durations and the number of movement units, *r* (87) = − .30, *p* = .004.

In general, infants showed variations in their reach durations. Infants did not employ a general strategy when obtaining the objects. Three infants only had reaches that were considered short, one infant had reaches that were considered medium, and four infants had reaches that were considered long. Eight infants had combinations of short and medium duration reaches. Eight infants had combinations of reaches that were of a medium and long duration, and six infants had combinations of short and long reach durations. Fifty‐seven participants reached for the objects using a combination of short, medium and long reaches.

With respect to movement units, reaches with very few movement units did not occur often. Reaches with 2–4 movement units were most prevalent; all 89 infants capable of reaching demonstrated reaches with 2–4 movement units. Thirteen infants demonstrated reaches that also contained less than 2 movement units in addition to reaches with 2–4 movement units. Twenty‐seven infants occasionally demonstrated reaches with movement units higher than 4 movement units in addition to the 2–4 movement units that everyone showed. This overview of movement units per participant showed that infants around 3 to 3.5 months of age do not yet consistently reach for objects with a particular number movement units, despite some consistency of the task across trials.

## DISCUSSION

The results demonstrate that reaching and grasping were similar for infants in all three conditions. Before and after training, there were no differences between the sticky mittens, observational and no training conditions in the time they spent reaching and grasping, the number of reaches, reach duration, number of movement units, grasp aperture, grasp aperture before touch, and grasp aperture after touch. General reaching ability did not differ between infants in the three conditions before and after training. These results show that, in the current study, the sticky mittens condition did not produce larger increases in reaching and grasping than the observational and no training conditions. At the same time, our results demonstrate changes in motor proficiency over a 2‐week period (pre‐test to post‐test), with an increase in the time infants spend reaching and gasping, the number of reaches, and reach duration. Grasp abilities at this age do not appear to change over a 2‐week period, as the grasp aperture does not change from pre‐test to post‐test. Moreover, infants fail to anticipate the object or react to it by changing their grasp.

These findings show that the training did not replicate in the current sample of Swedish infants. This pattern of results confirms the conclusion of a recent review suggesting mixed evidence for the claim that sticky mittens training encourages early motor development (van den Berg & Gredebäck, 2021). This further agrees with others who have cast doubt on the generalizability of training effects more broadly (Barnes et al., 2016; Stojanoski et al., 2020; Ballieux et al., 2016).

The suggestion that sticky mittens training can affect motor development has been extended to social perception studies. From an embodied cognition perspective (Juvrud & Gredebäck, 2020), manual behaviors are an essential part of infants' cognitive development (Gottwald et al., 2016; Gredebäck & Falck‐Ytter, 2015; Gredebäck & Daum, 2015). Through agentive manual interactions with the outside world, infants have been suggested to develop their social perception abilities. These agentive interactions can restructure representations of other people's goal actions (Gerson & Woodward, 2014a, 2014b; Sommerville et al., 2005), prompt innate but inaccessible knowledge of reaching (Skerry et al., 2013), facilitate observation of deviations of first‐hand reaching actions (Juvrud & Gredebäck, 2020), or activate the mirror neuron system (Gerson & Woodward, 2014a, 2014b; Nyström et al., 2011). In the context of this framework, the sticky mittens training has repeatedly been demonstrated to facilitate infants' understanding of reaching goals (Bakker et al., 2016; Gerson & Woodward, 2014a, 2014b; Sommerville et al., 2005) and the efficiency of an executed reach (Skerry et al., 2013). It is striking that these social perception effects exist in the absence of strong evidence of reaching and grasping in the current and other motor studies as suggested by van den Berg and Gredebäck (2021). The suggestion that sticky mittens training does not increase reaching and grasping does not line up with the idea of embodied cognition. However, some sticky mittens studies on social perception used only very brief sticky mittens training – too short to impact motor development – yet found significant effects on infant social perception (Sommerville et al., 2005). Therefore, future research is needed to assess which aspects of the sticky mittens procedure promote social perception and why (for a further discussion on the potential reasons for a social sticky mittens effect see Juvrud & Gredebäck, 2020).

Our results demonstrate that reach duration correlates with the number of movement units and that there is some stability in reaching. Long reaches consisted of more movement units whereas short reaches consisted of fewer movement units. Surprisingly, this relation is already present before the typical onset of mature reaching and grasping abilities of infants aged 5 months and older (von Hofsten, 1984). Although the results show variability between time points and within trials, this implies that the data have the precision to inform us about the manual ability of infants at this age.

However, before concluding, some caveats need to be highlighted. These results demonstrate little change between pre‐test and post‐test. Following the conclusions from van den Berg and Gredebäck (2021) this might indicate that the sticky mittens training is ineffective or that the training is effective but our design made it difficult to observe changes in reaching and grasping. To our knowledge, nothing could have interfered with our results, other than the limitations below. However, we cannot rule out other, unknown, factors that may have contributed. One limitation of our study is the lack of repeated home‐visits and assessments during the training period. Our study observed training fidelity in a small subset of participants via videoconference. These home‐visits likely served as reminders to the parent to foster motor skills during their daily interactions with the child. Further, repeated assessments of the child's reaching may also facilitate motor development. These two factors are likely additive to the effects of the sticky mittens training itself and may be critical for the success of parent‐guided motor training using sticky mittens. However, we cannot determine whether inclusion of these procedures would change the effectiveness of sticky mittens procedure. Due to the nature of the home‐visits, infants' learning process during training could not be assessed. We do not know how infants explored the object when it was stuck to their mitten, as we, unfortunately, do not have eye tracking or behavioral data during training. We are confident that training procedures were implemented correctly based on virtual home‐visits with some parents, completed training logs, and preliminary dose–response analyses that show no influence of training duration (see Supplementary Information). However, without empirically validating this for the entire sample it remains an open question.

With our kinematic reaching data, we can safely conclude that sticky mittens training does not improve the ability to reach or the microstructure of reaching. This is further confirmed by the 4‐step reaching task that has been used in many sticky mittens studies. The mittens allow infants to observe the outcome of a reach, which would make them more interested in reproducing this grasping outcome. Even in the absence of a positive effect on manual reaching and grasping behavior, infants may be developing new manual strategies that cannot be measured with the current tasks. While our results lead us to conclude that reaching and grasping skills (i.e., motor control) are not impacted by sticky mittens training, other developmental changes are possible and cannot be excluded based on the current results.

Another possibility is that the infants in this study were too mature to benefit from the training. On average, infants in this study were 2 weeks older (13 weeks at pre‐test) than infants in some prior sticky‐mittens studies that used the same parent‐implemented reaching training over a period of 2 weeks. These infants might have had more advanced reaching abilities, which would limit their learning process. However, from the first week of life until the age of 13 weeks, infants already show some reaching behaviors. Although there is within‐infant variability in reaching in this period, the average number of reaches does not increase in this period. A large increase in average reaching is observed when infants are 16 weeks old (von Hofsten, 1984). These typical abilities were also present at pre‐test in Libertus and Needham (2010). Moreover, other studies have successfully demonstrated sticky mittens effects with infants older than 13 weeks (Bakker et al., 2016; Gerson & Woodward, 2014a, 2014b; Skerry et al., 2013). Thus, it is unlikely that the infants' age could have negatively influenced training effects. This interpretation is supported by the current results. Adding age in days as a covariate did not alter the results in any way. However, we acknowledge that this study cannot rule out the effect of age on the effectiveness of the sticky mittens training.

In the kinematic reaching task, the removal of the object from the hand may have discouraged infants to reach for the objects in subsequent trials. It is possible that this is a reason why we do not observe differences in reaching and grasping behaviors between conditions. However, Figure 4 does not indicate that infants were discouraged during the task. In fact, most infants made several reaches and continued to reach throughout the session, making this alternative a less likely explanation for the results.

There is a possibility that the placement of sensors on the thumb and index finger may have interfered with an infant's ability to reach for and grasp an object, which may have reduced the differences between the three groups. However, each sensor was 2 mm thick and taped to the finger loosely to avoid any discomfort. Moreover, infants participated in a different task for 5 minutes prior to the current task. As this gave them time to get used to the sensor before the task began, it is unlikely that the sensor placement could have led to floor effects in the data. We also could not observe that infants were pre‐occupied with the sensors.

The surgical tape may also have prevented haptic feedback at pre‐and‐post‐test and during training. Haptic feedback has been demonstrated to be beneficial for reaching development, as it helps with practicing and fine‐tuning the skill (see Williams et al., 2015 and Corbetta et al., 2016 for a detailed account). The lack of feedback may have discouraged infants to reach for the objects or prevented learning during the training. Although the tape did not cover the entire finger, thus allowing some haptic feedback, the training effects might have been different with more haptic feedback. Unfortunately, this study does not allow us to assess the lack of haptic feedback in a way similar to Williams et al. (2015), as this study attempts to replicate only the original sticky mittens training.

The results were based on a relatively small sample size. Although the current sample is larger than previous sticky mittens studies, it is possible that a larger sample will have more power to demonstrate facilitated training effects. Considering the inconsistencies in sticky mittens studies reported by van den Berg and Gredebäck (2021), it is important that negative findings are replicated as well as positive findings in order to examine whether these results are generalizable. Note that the quality of a study is not measured solely by whether it is pre‐registered, as outlined above other factors in the training could have affected the current study. Before concluding that sticky mittens do not enhance manual motor development, reaching and grasping, this study needs to be replicated as well.

Before concluding, there are also a series of potentially important cultural difference between this study and prior work that should be considered when interpreting the results.

First, parents in different cultures might differ in their beliefs of the importance of motor stimulation and the way of interacting with infants. For example, while Cameroonian mothers typically prioritize motor independence and milestone acceleration when raising infants, German mothers prioritize the relationship with infants (Keller, Yovsi, & Voelker, 2002). If similar differences exist between this and prior studies, it is possible that parents' beliefs about childhood and parenthood differ between this sample and prior work.

Secondly, it is possible that the extended Swedish parental leave may have contributed to the null‐effects, as parents may have more opportunities to engage in motoric interactions with their child. As such, the long parental leave policy may have overridden the sticky mittens effect, such that infants were too skilled at the start of the training. However, given that the process of learning to reach is infant‐directed and internally driven (von Hofsten, 2004) it is unlikely that potential extra interactions may be sufficient to interfere with the natural learning process. Moreover, European 3‐month‐old infants have been found to spend less time in physical proximity to parents compared to infants in other parts of the world (Keller et al., 2005), thus suggesting that long parental leave policies may not necessarily result in an equal enhancement in physical interactions. However, this possibility cannot be ruled out with the current data and therefore future work needs to examine the effects of long parental leave on infant motor development and its effects on motor training.

Third, it is possible that cultural differences exist between Swedish and the U.S. infants in the onset and development of reaching and that this difference can account for the results. Infants from several African cultures have been found to exhibit motor abilities, such as sitting, at an earlier age than infants in Western, educated, industrialized, rich and democratic populations (WEIRD; Karasik et al., 2015; Super 1976). Similar cultural differences between infants are also found within WEIRD populations. Dutch infants (15 months, Oudgenoeg‐Paz et al., 2016) have been found to have a later walking onset than U.S. infants (12 months, Adolph et al., 2011). If similar differences exist in reach onset between Swedish and U.S. infants, the exact timing of when the training is offered might be important in order to observe reaching and grasping improvements. In both Sweden and the U.S., reach onset has been reported between 4 and 6 months with large variations between infants (von Hofsten, 1991; Thelen et al., 1993; White et al., 1964). We believe this interpretation is not the most likely interpretation of results as age in days did impact the results, but more work is needed in order to fully explore this interpretation.

Fourth, American parents have been found to play with their 3‐month‐old infant at a faster pace compared to Swedish parents (Hedenbro, Shapiro, & Gottman, 2006). It is possible that similar differences in pace of play may have contributed to the difference in sticky mittens findings between U.S. infants and the European infants in the current study. An important element of the training is that the infant is allowed 10 s to explore the toy. However, parents still have the freedom to make judgement‐calls with regards to the timing of retrieval of the toy. The U.S parents may retrieve the toy faster in order to match their usual communication style, which may be necessary for the sticky mittens training to work. This could explain why the training results did not generalize to the current study, as these parents may retrieve the toy at a slower pace. Future work may need to focus on investigating cultural differences in the execution of the training and whether the training may need to be adjusted in different cultures.

With these caveats in mind we carefully propose an alternative interpretation, one that is in line with van den Berg and Gredebäck (2021), namely that the amount of sticky mittens training provided in the paradigm is not enough to match the amount of experience infants naturally receive and that this is true for all the above cited sticky mittens studies. As described earlier, the emergence of motor abilities requires massive motor experiences and often starts in the womb (Zoia et al., 2007). The sticky mittens training typically consists of 140 minutes of exposure to reaching experiences, while natural motor experiences require several months for reaching and grasping to emerge. By the age of three months, infants have acquired a considerable amount of reaching experiences and they are very close to showing the first signs of goal‐directed reaching. From this perspective, it is likely that the sticky mittens training may not provide them with sufficient additional experiences to expedite reach emergence and strong motor patterns compared to the internally motivated learning they have been engaged in for months. It has previously been suggested that findings in previous studies may also have been caused by other factors in the training, such as increased parental encouragement, context, exposure to toys, or interaction with the experimenter (Libertus & Needham, 2010). Future work will need to examine this more closely.

In summary, the current study failed to generalize the common sticky mittens effect to this Swedish sample, using movement dynamics in this sample of 3‐month‐old infants. Neither the ability to reach nor the microstructure of reaching and grasping were affected by the training. To understand the sticky mittens training better, the role of parental behavior will require further investigation. More work is needed to understand if there are unknown mechanisms that explain why the current study could not demonstrate facilitating effects while previous studies did. It is imperative that the role of culture is further examined, as it is plausible that the sticky mittens training may have to be adjusted for different cultures in order to generalize to other infant samples. Future research will need to examine how the sticky mittens training affects social perceptive and attentional abilities in the absence of motor improvements.

## Supporting information


**Figure S1** Performance on each variable from pre‐test to post‐test for individual infants. The dose–response correlation is indicated for all variables for both the sticky mittens condition and observational condition.
