# Supplementary material for: A Pre‐registered sticky mittens study: active training does not increase reaching and grasping in a swedish context
Source: Child Dev. 2022 Sep 1;93(6):e656–71. doi: 10.1111/cdev.13835 (PMC9826026; doi:10.1111/cdev.13835)
Supplement: Supplementary file 1 — Figure S1 Performance on each variable from pre‐test to post‐test for individual infants. The dose–response correlation is indicated for all variables for both the sticky mittens condition and observational condition. [file CDEV-93-e656-s001.pdf]

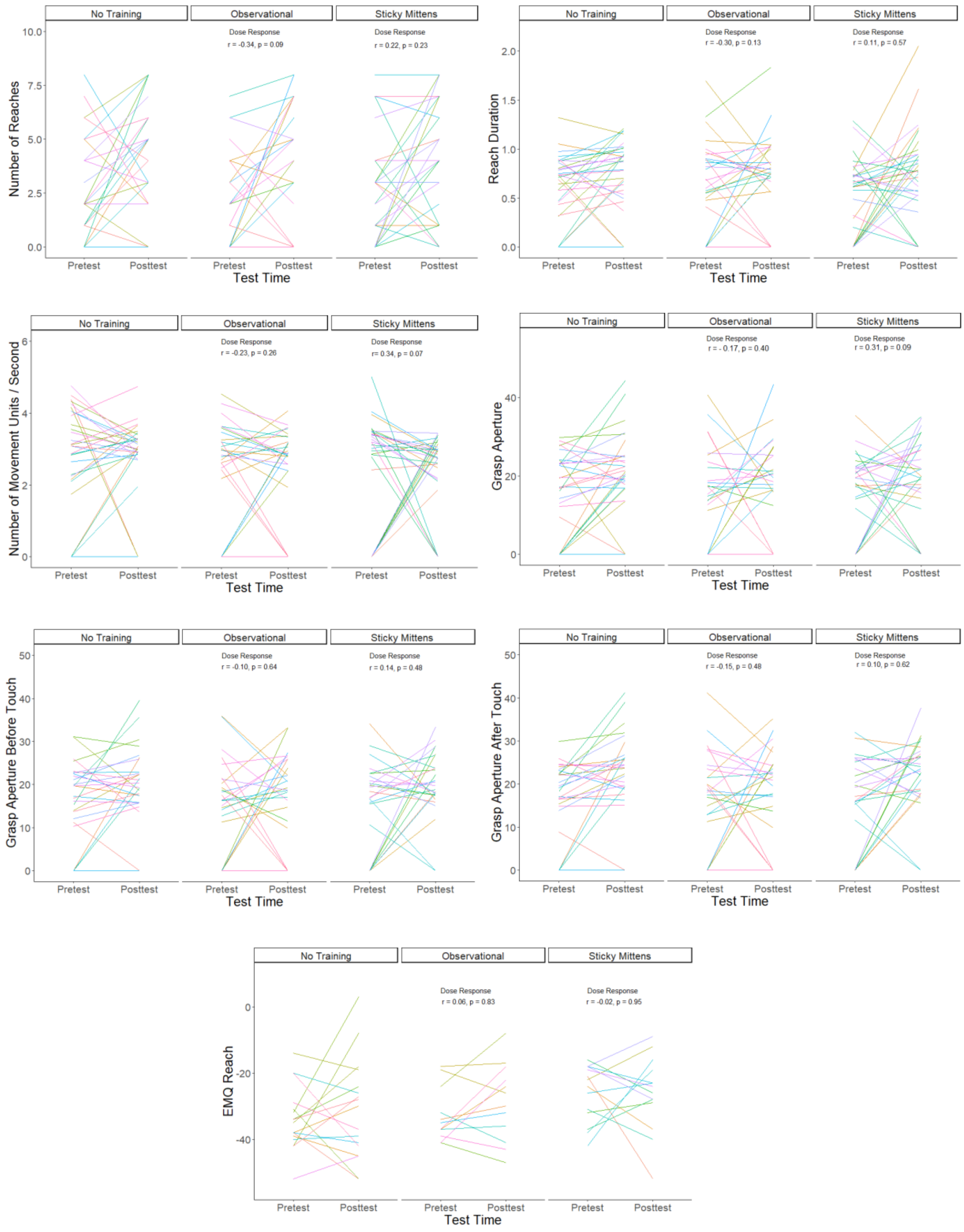
No Training
Observational
Sticky Mittens
Dose Response
r = -0.34, p = 0.09
r = 0.22, p = 0.23
Number of Reaches
Reach Duration
r = -0.30, p = 0.13
r = 0.11, p = 0.57
Number of Movement Units / Second
r = -0.23, p = 0.26
r= 0.34, p = 0.07
Grasp Aperture
r = - 0.17, p = 0.40
r = 0.31, p = 0.09
Grasp Aperture Before Touch
r = -0.10, p = 0.64
r = 0.14, p = 0.48
Grasp Aperture After Touch
r = -0.15, p = 0.48
r = 0.10, p = 0.62
EMQ Reach
r = 0.06, p = 0.83
r = -0.02, p = 0.95
Pretest
Posttest
Test Time

Figure S1. Performance on each variable from pre-test to post-test for individual infants. The dose-response correlation is indicated for all variables for both the sticky mittens condition and observational condition.
